# Investigation of Electrospark Sintering of Composites of SiC–TiC, SiC–VC Systems

**DOI:** 10.3390/ma18030508

**Published:** 2025-01-23

**Authors:** Vyacheslav Ivzhenko, Ruslan Vovk, Edvin Hevorkian, Tamara Kosenchuk, Volodymyr Chyshkala, Volodymyr Nerubatskyi, Vadym Cherniavskyi, Natalia Shamsutdinova

**Affiliations:** 1 V. Bakul Institute for Superhard Materials of the National Academy of Sciences of Ukraine, 2 Avtozavods’ka St., 04074 Kyiv, Ukraine; ivv@ism.kiev.ua (V.I.); avl@ism.kiev.ua (T.K.); vadikv13@gmail.com (V.C.); 2 Department of Reactor Engineering Materials and Physical Technologies, V. N. Karazin Kharkiv National University, 4 Svobody Sq., 61022 Kharkiv, Ukraine; rvvovk2017@gmail.com (R.V.); vchishkala@ukr.net (V.C.); 3 Department of Mechanical Engineering and Automation, University of Life Sciences in Lublin, 28 Głęboka St., 20-612 Lublin, Poland; 4 Department of Electrical Energetics, Electrical Engineering and Electromechanics, Ukraine State University of Railway Transport, 7 Feuerbach Sq., 61050 Kharkiv, Ukraine; nevlpa9@gmail.com; 5 Plasma Tech LLC, 13 Khmelnytske Shosse, 21036 Vinnytsia, Ukraine; shamsut.natalka49@gmail.com

**Keywords:** silicon carbide, titanium carbide, vanadium carbide, electrօspark sintering, sintering temperature

## Abstract

The kinetic regularities of the electrօspark sintering of SiC–TiC, SiC–VC composites at a pressure of 45 MPa and the temperatures of 1900 and 2000 °C have been established. At the first stage of the composite compaction process, the addition of TiC, VC impurities in the amount of 20 vol.% to silicon carbide with a dispersion of 2 μm increases the compaction rate by 1.3 and 1.1 times, respectively, and the addition of Ti, V carbides in the amount of 40 vol.% increases the compaction rate by 1.7 and 1.2 times, respectively. At the second stage of the compaction process, when Ti, V carbides are added in the amount of 40 vol.%, the compaction increases from 70% in silicon carbide to 99.9% in the 60SiC–40TiC composite and 91.2% in the 60SiC–40VC composite. Solid-phase sintering in composites with an admixture of titanium carbide is better than in composites with an admixture of vanadium carbide due to an increase in interaction at the phase boundaries: the interaction zone increases from ~1.0 μm at the boundaries of silicon carbide and vanadium carbide grains to ~1.5 μm at the boundaries of silicon carbide and titanium carbide grains.

## 1. Introduction

Silicon carbide has significant potential for the manufacture of high-temperature, wear-resistant, and corrosion-resistant materials due to its high hardness, strength, high creep resistance, and significant oxidation resistance. For example, the addition of SiC increases the thermal conductivity of cutting inserts and reduces diffusion and adhesive wear during machining at high cutting speeds [1].

Pressure-sintering technology (hot pressing) is widely used in the powder metallurgy of refractory compounds to produce materials with minimal porosity. By changing the process parameters, researchers can obtain materials with varying porosity and structure [2,3]. For this purpose, the kinetics of the densification of material samples during sintering is studied based on the dependence of shrinkage (Δ*l*/*l*) on temperature, pressure, and holding time.

Obtaining dense products by hot pressing from technically pure silicon carbide powders is impossible due to their low plasticity even at a temperature of 2000 °C [4]. The densification of these powders occurred only at temperatures close to the dissociation temperature of silicon carbide (2700 °C) and with the introduction of impurities that form a liquid phase [5]. The use of pressure sintering allows obtaining dense SiC materials with the highest physical and mechanical properties [6]. Currently, this is the best industrial technology for producing silicon carbide products that operate in difficult operating conditions.

Limited literature exists on the impact of refractory metal impurities, nitrides, carbides, borides, and silicides on the densification of silicon carbide without forming a liquid phase. The effect of amorphous boron, molybdenum, and molybdenum disilicide impurities on the densification of silicon carbide at temperatures of 2140–2170 °C was investigated [5]. It has been shown that an admixture of amorphous boron in an amount of 10–20 wt.% is the most effective for pressing dense samples (porosity 3–4%) from powders with a particle size of 60 μm at a pressure of 100 MPa. The effect of carbon and boron on the sintering of silicon carbide with a specific surface area of 15.7 m^2^/g was investigated in [7,8]. It has been found that carbon impurities (without boron) contribute to slight densification. A carbon content of 1.5–3.0 wt.% is effective in retarding the growth of silicon carbide grains. The effect of aluminum nitride on the densification of silicon carbide was investigated in [9]. Due to solid-phase interaction, a porosity of 2–3% can be achieved. Titanium carbide and titanium nitride are used as strengthening phases in the materials of the SiC–Al_2_O_3_–Y_2_O_3_ system [10,11,12,13,14]. The studies conducted in [15,16] experimentally confirm the idea that was formulated in the sentence “The presence of TiC particles changes the morphology of silicon carbide grains; they complicate the growth of elongated crystallites and ensure the formation of more equiaxed grains”.

Flash sintering [17,18] is a new process for manufacturing consolidated materials that provides rapid heating, saves energy and time, and densifies ceramics more effectively compared to more traditional sintering methods. Electrospark sintering can be a tool not only for densifying materials but also for designing microstructures. The speed of the process allows for the creation of new temperature profiles that can suppress atomic diffusion and enable the formation of thermodynamically metastable materials and microstructures. The unidirectionality of the electric field is a common feature in most assisted sintering methods and can also be reflected in the microstructure of the sintered materials. When using a constant electric field, strongly directional (polarity dependent) effects can be obtained as a result of the following: (I) thermal gradients generated in the sample; (II) the Peltier/Thomson effect [19]; (III) electromigration [20]; (IV) electrochemical reduction [21]. These directional effects can be used to develop textured or functional gradient microstructures [22]. The developed flash spark plasma sintering (FSPS) process allows for the simultaneous densification and texture in SiC ceramics [23], and in a direction parallel to the loading direction. The texture forms as a result of the temperature gradient in the sample, which arises from the higher electrical resistance of SiC compared to that of graphite punches.

The purpose of this work is to study the kinetics of sintering and the regularities of the formation of the structure of dense materials based on silicon, titanium, and vanadium carbides obtained by the method of electrօspark sintering at a pressure of 45 MPa.

## 2. Materials and Methods

For this study, α–SiC powder of the M2 grade produced by the Zaporizhzhya Abrasive Plant (Zaporizhzhya, Ukraine) with an average particle size of 2 μm was used. The starting powder contained ~98% SiC and no more than 0.1% Fe, 1.5% O, and 0.4% C. TiC powder with an average particle size of 4 μm and VC powder with an average particle size of 6 μm were used as additives. The powders were mixed in a ball mill in a humid environment using grinding media made of hot-pressed silicon carbide for 24 h.

The samples were obtained by electric spark sintering in graphite molds at temperatures of 1900, 2000 °C under a pressure of 45 MPa for 15–45 min. The current was 5000A, the voltage was 5 V, and the heating rate was 300 degrees/min. Sintering was carried out in a vacuum of 10^−2^ mm Hg.

During electrospark sintering, the relative change in size (height) of the sample was monitored depending on the time of the technological process. The samples were measured and weighed before and after sintering.

There are a large number of empirical equations to describe the kinetics of densification. A regression analysis of the kinetic equations showed that the sintering process is described with sufficient accuracy by the Avrami–Erofeev rate equation for topochemical reactions. It can be applied to sintering if densification is considered as a void disappearance reaction:

*F* = 1 − exp(−*k*·*t^n^*), (1)

where *F* is the degree of compaction, which is equal to *F* = (*l_o_* − *l_t_*)/(*l_o_* − *l_d_*); *l_o_*, *l_t_*, *l_d_* is, respectively, initial, current, and final (full) shrinkage, *t* is time, *k* is the compaction rate constant, and *n* is the compaction time constant.

The kinetic parameters of the compaction process k and n can be determined using the logarithmic form of Equation (1):

ln(1 − *F*) = −*k*·*t^n^*, (2)

ln[−ln(1 − *F*)] = ln*k* + *n*·ln*t*, (3)

The graphs are plotted in the coordinates ln[−ln(1 − *F*)] and ln*t*. These are straight, broken lines characterized by different angles of inclination and, accordingly, different values of *n* and *k*. On this basis, the compaction process can be divided into stages that differ in mass transfer mechanisms [24].

The density and porosity of the material were calculated using the method regulated by DSTU EN ISO 3369:2014 [25].

The material samples were examined at High Energy Technologies LLC (Vinnytsia, Ukraine). This study was conducted using a Tescan Vega 3 SBH EP scanning electron microscope (Tescan Brno s.r.o., Brno, Czech Republic). Microstructural studies were carried out with an accelerating voltage of 30 kV in the BSE (reflected electrons) and SE (secondary electrons) modes at different magnifications. The determination of the chemical composition was performed using mapping methods at an accelerating voltage of 30 kV, point analysis, and the scanning of the sample surface with an area of 0.09–0.25 mm^2^. The Bruker Quantax 610M energy dispersive spectrometer (Company “Bruker Optik GmbH”, Ettlingen, Germany), mounted on a Tescan Vega 3 SBH EP scanning electron microscope, allows the detection of elements from B (z = 4) to Am (z = 95).

Vickers hardness measurements *H_V_* (at a load of 50 N) were performed on a Matsuzawa MXT70 digital microhardness tester. The pyramid imprint was studied on an NU-2E optical microscope manufactured by Carl Zeiss (Oberkochen, Germany) at 750× magnification. The determination of crack resistance (fracture toughness *K*_1*c*_) was carried out using the Evans–Charles method based on the length of radial cracks from the corners of the Vickers indenter imprint.

## 3. Theoretical Background

The use of lightweight materials like silicon carbide (SiC) and boron carbide (B_4_C) in military applications is crucial for enhancing the performance and effectiveness of armor systems. Boron carbide, with its low density of 2.51 kg/m^3^ and high modulus of elasticity (480 GPa), provides excellent ballistic protection while minimizing weight. This allows for greater mobility and agility in military operations. Silicon carbide, though slightly denser at 3.21 kg/m^3^, still offers impressive mechanical properties with a modulus of elasticity of 432 GPa. Its lower cost and easier processing compared to B_4_C make it a viable alternative for armor applications, especially when budget constraints are a concern [26,27]. The combination of these materials’ properties allows for effective energy dispersion from ballistic impacts. Consequently, while B_4_C may be the superior material in terms of ballistic performance, the industry often utilizes a combination of these ceramics to balance performance, cost, and manufacturability in armor systems. For continued advancement, ongoing research into improving the processing techniques for boron carbide and exploring hybrid materials may provide further options for military applications, ensuring both cost-effectiveness and superior protection. The main common feature of nonmetallic carbides (SiC, B_4_C) is their strong covalent atomic bonding. In the case of SiC, 78% of the total SiC energy bonding is purely covalent, characterized by the strong and stable sp^3^ high-energy configurations [28]. Silicon carbide is essentially a diamond crystal in which half of the carbon atoms are replaced by silicon atoms having less stable sp^3^ configuration [29]. This predominant covalent character associated with very strong atomic bonding confers remarkably high mechanical properties and thermodynamic stability to these ceramics. The strong covalent bonding in nonmetallic carbides such as silicon carbide (SiC) and boron carbide (B_4_C) is indeed a key feature that contributes to their exceptional mechanical properties and thermal stability. This covalent bonding results in a robust and stable structure, allowing these materials to withstand high temperatures and mechanical stresses. In SiC, the predominant sp^3^ hybridization creates a strong covalent network, which is similar to diamond, but with silicon atoms partially substituting for carbon. This configuration enhances its hardness and thermal conductivity while providing significant resistance to wear and deformation. As mentioned, the covalent nature of SiC bonding accounts for about 78% of the total bonding energy, underscoring its strong and durable characteristics. The remarkable mechanical properties and thermodynamic stability of SiC and B_4_C make them suitable for demanding applications, including those in military and aerospace fields, where material performance under extreme conditions is critical. As research continues, the further understanding and manipulation of these bonding characteristics may lead to the development of even stronger and more resilient ceramic materials. However, the high rigidity of atoms bonding in the lattice of these ceramics also causes their low diffusion mobility. For example, the activation energy of C diffusion in SiC is extremely high (713.7 kJ/mole at 1850–2180 °C), which is 2–3 times higher than in metallic carbides. The activation energy for Si diffusion is even higher (910.2 kJ/mol at 2010–2274 °C). The low atom mobility makes this type of ceramic very hard to sinter. Currently, SiC, B_4_C-based materials are consolidated by hot pressing and HIP using different sintering aids [30,31,32,33,34,35].

The usual additives for SiC are metal oxides (Al_2_O_3_ and rare-earth oxides) and simultaneous additions of B and C [36]. Sintering temperatures are in the range of 1850–2000 °C for oxide additives and in excess of 2000 °C for B and C. The most frequent additives to enhance the sintering of B_4_C are liquid-forming metals, such as Al, Ti, Ni, and Fe, or oxides (Al_2_O_3_ and Y_2_O_3_) [37,38,39,40,41,42]. For the sintering of B_4_C, transition metal borides and carbides (of Ti, V, Cr, W) are used in quantities usually exceeding 10 wt% [43,44,45]. Some additives hindering grain growth were found to also be effective in improving mechanical properties. For instance, Al_2_O_3_ and Y_2_O_3_ are favorable additives to SiC ceramics, providing a bending strength of 625 MPa and a fracture toughness of 7.5 MPa·m^1/2^ [46]. For example, the Ni addition in TiB_2_ is reported to result in a bending strength of 670 MPa and a fracture toughness of 6.4 MPa·m^1/2^ [39]. The flexural strength reaches 630 MPa and a fracture toughness of 3.5 MPa·m^1/2^ in B_4_C using a CrB_2_ additive [45]. These mechanical properties are for dense ceramics with 2–5 μm grain size. Further improvement may be achieved by sintering without additives and reducing the grain size. Most of the additives enhance diffusion by a liquid-phase mechanism, but the additive-rich (glassy) phases remain at grain boundaries in the final sintered body. To further improve mechanical properties, mainly strength and fracture toughness, it is well known that high-purity, fine-grained, and completely dense ceramics are required. Attempts were made to densify these ceramics without sintering aids. For instance, Allegro et al. reached a density of 84% by hot pressing SiC at 2350 °C under a pressure of 60 MPa [47]. Generally, the pressure range applied during hot pressing is too low to induce any plastic deformation of SiC. B_4_C without aids was hot pressed to 88% density at 2000 °C for 1 h under 30 MPa pressure [26]. Long-time sintering causes considerable grain growth. In addition, the application of ultrahigh pressure makes this technique expensive, and, at the same time, there are severe size limitations. The investigation into the sintering of hard-to-sinter nanocrystalline ceramics like silicon carbide (SiC) and boron carbide (B_4_C) represents a significant advancement in materials science. Achieving full density in these ceramics, which traditionally required liquid-forming additives, points to new possibilities for enhancing their processing techniques. The focus on direct intergranular bonding in the sintering process is particularly noteworthy, as this can lead to improved mechanical properties and thermal stability without relying on additional liquid phases. Understanding the mechanisms that govern the sintering behavior of these ceramics can provide critical insights into optimizing their densities and microstructures and potentially reducing production costs. The principles derived from this investigation may indeed be applicable to other ceramics with strong covalent bonding, which typically exhibit similar challenges in densification. By developing methods that allow the successful consolidation of such materials, researchers can expand the range of applications for high-performance ceramics in various fields, including aerospace, defense, and advanced engineering.

Future research and development efforts could focus on exploring different sintering atmospheres, temperature profiles, and innovative additives (such as nanomaterials) that may further enhance the properties and processing of these difficult-to-sinter ceramics. This approach not only promises broader application ranges but also paves the way for the development of new ceramic materials with tailored properties for specific end-use applications.

## 4. Results and Discussion

From the initial powder mixtures of the composition 80 vol.%SiC–20 vol.%TiC, 60 vol.%SiC–40 vol.%TiC, 80 vol.%SiC–20 vol.%VC, 60 vol.%SiC–40 vol.%VC, silicon carbide with a dispersion of 2 μm at temperatures of 1900 and 2000 °C, holding times of 15–45 min, by the method of electrօspark sintering, samples of materials with a diameter of 11 mm were produced. The pressure was applied at a temperature of 1000 °C, increased to 45 MPa in 3 min and reduced to 0 MPa 3 min after the start of cooling.

As a result of the experiments, the sintering kinetics of SiC–TiC, SiC–VC, and silicon carbide composites with a dispersion of 2 μm at a pressure of 45 MPa were investigated, namely the following: the dependences of the sintering parameter on the sintering time were constructed for composites with different contents of TiC and VC at temperatures of 1900, 2000 °C, using the Avrami–Erofeev equation, the sintering kinetic constants *k* (characterizing the densification rate) and *n* (characterizing the densification process time) were determined at a pressure of 45 MPa at temperatures of 1900 and 2000 °C.

**Figure 1:**
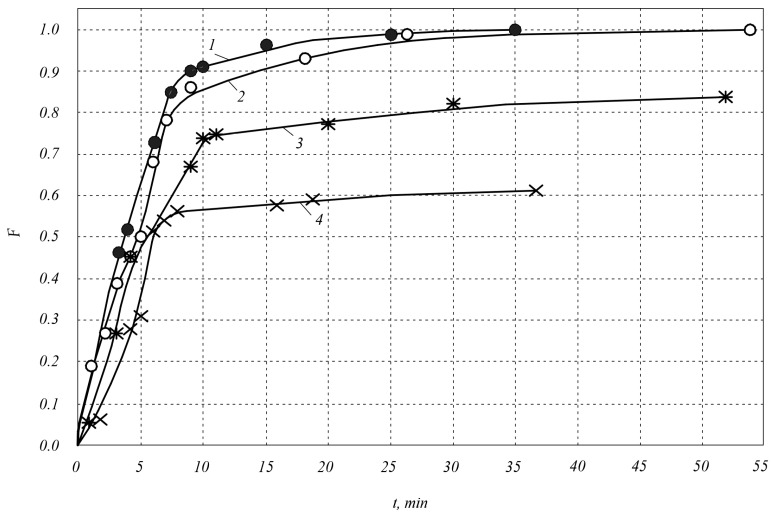
Dependence of the sintering parameter *F* on the holding time *t* during electrospark sintering of SiC–TiC composites: 1—composition 60 mol.%SiC–40 mol.%TiC at a sintering temperature of 2000 °C (●); 2—composition 60 mol.%SiC–40 mol.%TiC at a sintering temperature of 1900 °C (○); 3—composition 80 mol.%SiC–20 mol.%TiC at a sintering temperature of 2000 °C (ӿ); 4—silicon carbide with a dispersion of 2 μm (×).

Figure 1 shows the dependence of the sintering parameter F on the sintering time *t* during the sintering of SiC–TiC composites of the compositions 80 mol.%SiC–20 mol.%TiC; 60 mol.%SiC–40 mol.%TiC; silicon carbide with a dispersion of 2 μm at temperatures of 1900 and 2000 °C.

**Figure 2:**
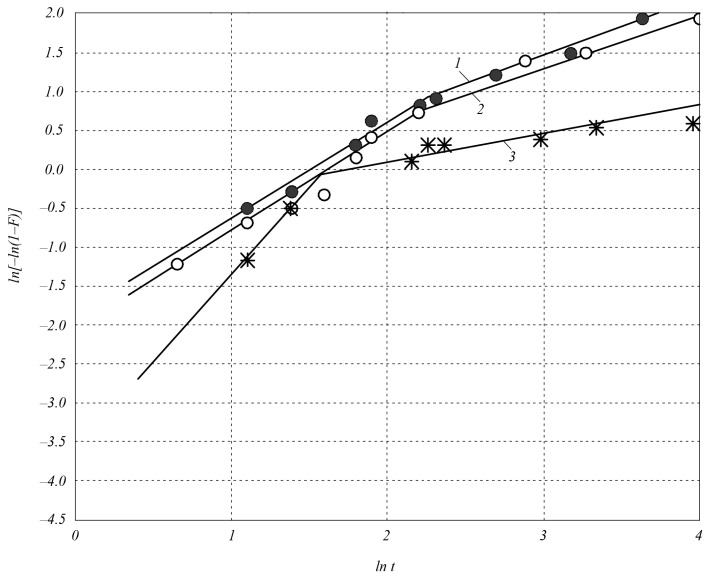
Dependence of the parameter ln[–ln(1–*F*)] on ln*t* during electrospark sintering of SiC–TiC composites: 1—composition 60 mol.%SiC–40 mol.%TiC at a sintering temperature of 2000 °C (●); 2—composition 60 mol.%SiC–40 mol.%TiC at a sintering temperature of 1900 °C (○); 3—composition 80 mol.%SiC–20 mol.%TiC at a sintering temperature of 2000 °C (ӿ).

Figure 2 shows the dependences of the parameter ln[−ln(1 − *F*)] on ln*t* during the sintering of SiC–TiC composites with compositions of 80 mol.%SiC–20 mol.%TiC; 60 mol.%SiC–40 mol.%TiC at temperatures of 1900 and 2000 °C.

**Figure 3:**
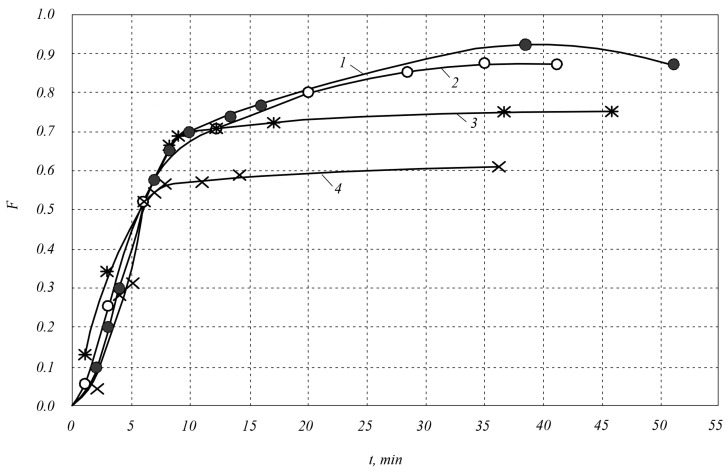
Dependence of the sintering parameter *F* on the holding time *t* during electrospark sintering of SiC–VC composites: 1—composition 60 mol.%SiC–40 mol.%VC at a sintering temperature of 2000 °C (●); 2—composition 60 mol.%SiC–40 mol.%VC at a sintering temperature of 1900 °C (○); 3—composition 80 mol.%SiC–20 mol.%VC at a sintering temperature of 2000 °C (ӿ); 4—silicon carbide with a dispersion of 2 μm (×).

Figure 3 shows the dependence of the sintering parameter *F* on the sintering time *t* during the sintering of SiC–VC composites with compositions of 80 mol.%SiC–20 mol.%VC; 60 mol.%SiC–40 mol.%VC; silicon carbide with a dispersion of 2 μm at temperatures of 1900 and 2000 °C.

**Figure 4:**
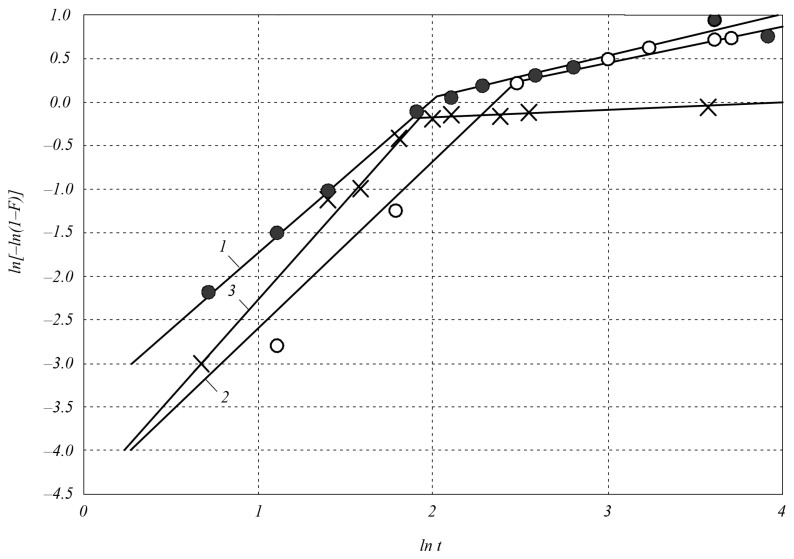
Dependence of the parameter ln[−ln(1 − *F*)] on ln*t* during electrospark sintering of SiC–VC composites: 1—composition 60 mol.%SiC–40 mol.%VC at a sintering temperature of 2000 °C (●); 2—composition 60 mol.%SiC–40 mol.%VC at a sintering temperature of 1900 °C (○); 3—silicon carbide with a dispersion of 2 μm (×).

Figure 4 shows the dependences of the parameter ln[−ln(1 − *F*)] on ln*t* during the sintering of SiC–VC composites with a composition of 60 mol.%SiC–40 mol.%VC; silicon carbide with a dispersion of 2 μm at temperatures of 1900 and 2000 °C.

The calculated values of kinetic constants during electrospark sintering *n*, *k* and the obtained porosity *P* of the composites are presented in Table 1.

As can be seen from Table 1, the porosity of composites with VC additives is higher than with TiC. At the same time, increasing the content of carbide additives contributes to the better compaction of composites, while the sintering temperature does not affect them significantly.

From Figure 1 and Figure 3, it is clear that the compaction process can be divided into two stages, which differ in the mass transfer mechanism. The first stage is characterized by rapid compaction: it accounts for ~71–91% of the total compaction. According to the literature data [5], shrinkage occurs as a result of the rotation and rearrangement of grains due to sliding in the contact zone. The second stage is characterized by a low compaction rate and its negative acceleration.

An analysis of the research results shows that at the first stage of the process of the compaction of composites, adding impurities TiC and VC in an amount of 20 vol.% to silicon carbide with a dispersion of 2 μm increases the compaction rate by 1.3 and 1.1 times, respectively. Adding to silicon carbide titanium carbide and vanadium carbide in an amount of 40 vol.% increases the compaction rate by 1.7 and 1.2 times, respectively.

At the first stage, the compaction process time (constant *n*) is shorter in the 60SiC–40TiC composite (*n* = 1.3 at *T* = 1900 and *T* = 2000 °C) and in the 80SiC–20TiC composite (*n* = 2.0 at *T* = 2000 °C) than in the 60SiC–40VC composite (*n* = 1.7 at *T* = 1900 °C, *n* = 1.8 at *T* = 2000 °C) and in silicon carbide (*n* = 2.1 at *T* = 2000 °C). At the same time, the densification rate constant k is characterized by high values during the densification of the 60SiC–40TiC composite (*k* = 0.11 at *T* = 1900 °C, *k* = 0.14 at *T* = 2000 °C) and insignificant values during the densification of the 80SiC–20TiC composite (*k* = 0.04 at *T* = 2000 °C), the 60SiC–40VC composite (*k* = 0.01 at *T* = 1900 °C, *k* = 0.03 at *T* = 2000 °C), and silicon carbide (*k* = 0.01 at *T* = 2000 °C).

As a result, at the first stage of sintering, the maximum densification is achieved in the 60 SiC–40 TiC composite sintered at *T* = 2000 °C, namely ~91% of the final one. In the 60 SiC–40 TiC composite sintered at *T* = 1900 °C, the densification at the first stage of sintering is ~86%, in the 80 SiC–20 TiC composite sintered at *T* = 2000 °C, it is ~80%. In the 60 SiC–40 VC composite sintered at *T* = 1900 and 2000 °C, the maximum densification is ~79% of the final one, in the 80 SiC–20VC composite sintered at *T* = 2000 °C, it is ~86%. In silicon carbide sintered at *T* = 2000 °C, the maximum densification is ~92% of the final one.

At the second stage of the densification process, adding 40 vol.% Ti and V carbides with a dispersion of 2 μm to silicon carbide significantly increases densification; namely, with optimal parameters of electrospark sintering under a pressure of 45 MPa, the maximum densification increases from ~70% in silicon carbide to 99.9% in the 60SiC–40TiC composite and 91.2% in the 60SiC–40VC composite. When adding Ti, V carbides to silicon carbide in an amount of 20 vol.%, the maximum densification is insignificant: it increases to 88% in the 80SiC–20TiC composite and to 75% in the 80SiC–20VC composite. At the second stage of the process, the densification rate constant *k* for the 60SiC–40TiC composite is 0.5 and does not depend on the sintering temperature. For the 60SiC–40VC composite, it is 0.4 and also does not depend on the sintering temperature. The time constant of the densification process n in the 60SiC–40TiC composite is 0.6 at a sintering temperature of 1900 °C; when the temperature is increased to 2000 °C, it increases to 0.7; when the titanium carbide content in the initial charge decreases to 20 vol.%, it decreases to 0.4. The constant *n* in the 60SiC–40VC composite is 0.5, and no effect of temperature on it was detected in the range of 1900–2000 °C. The time constant of the densification process *n* during the sintering of silicon carbide at the second stage of the process is much smaller and is 0.1.

**Figure 5:**
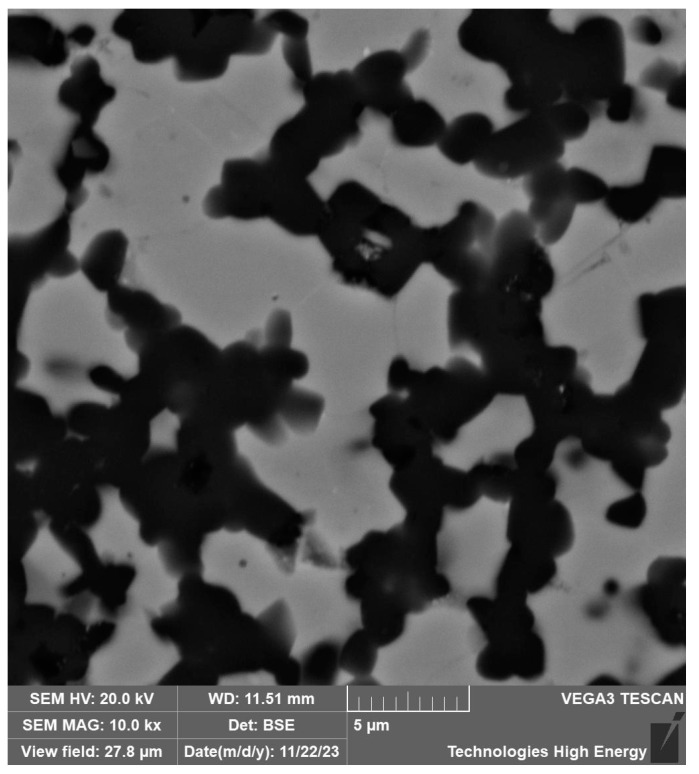
Microstructure of the surface of the 60SiC–40TiC composite section obtained at a sintering temperature of 2000 °C, a pressure of 45 MPa, and a holding time of 30 min.

**Figure 6:**
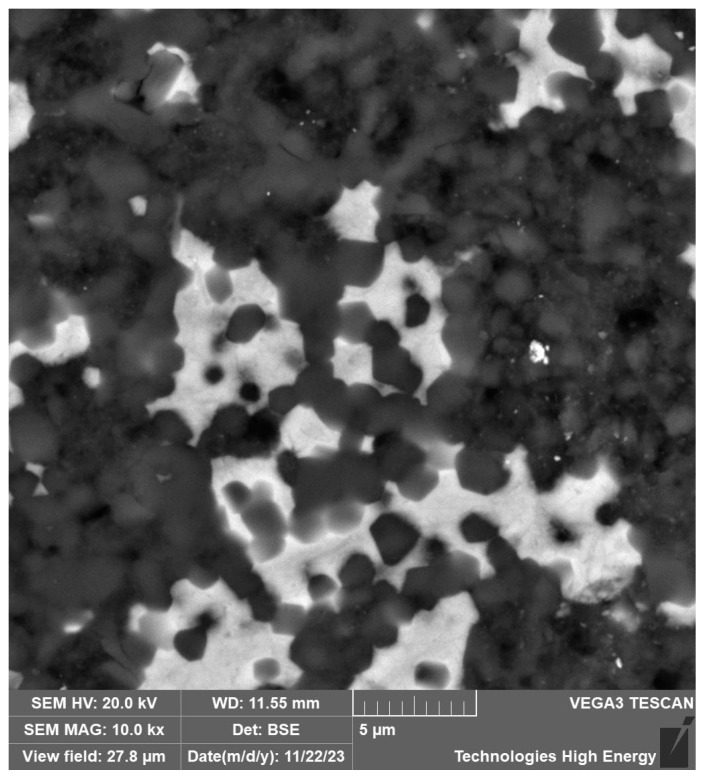
Microstructure of the surface of the 60SiC–40VC composite section obtained at a sintering temperature of 2000 °C, a pressure of 45 MPa, and a holding time of 30 min.

The samples of the obtained materials were studied using structural and micro-X-ray spectral analysis methods. The structure of the 60SiC–40TiC material consists of the gray grains of the matrix phase of silicon carbide and with sizes ranging from 1 to 7 μm (Figure 5). The structure of the 60SiC–40VC material also consists of the gray grains of the matrix phase of silicon carbide and the light inclusions of vanadium carbide with a size of 2–10 μm (Figure 6).

**Figure 7:**
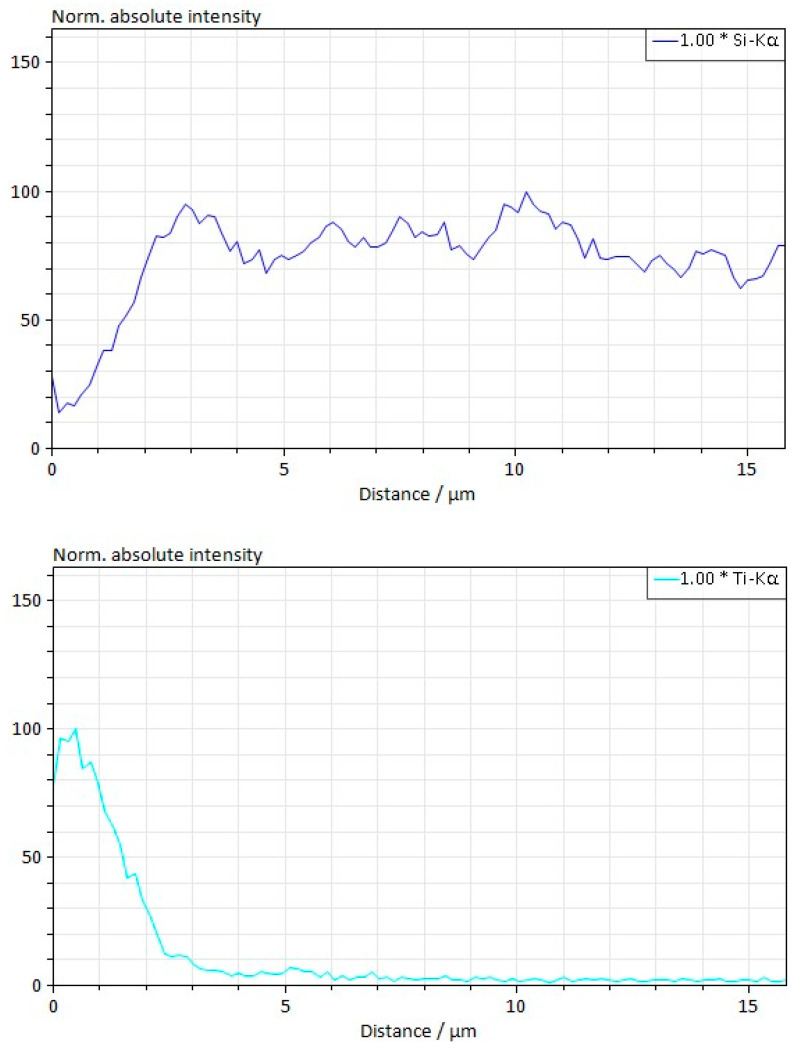
Zone of interaction between grains in the 60SiC–40TiC composite obtained at a sintering temperature of 2000 °C, a pressure of 45 MPa, and a holding time of 30 min.

**Figure 8:**
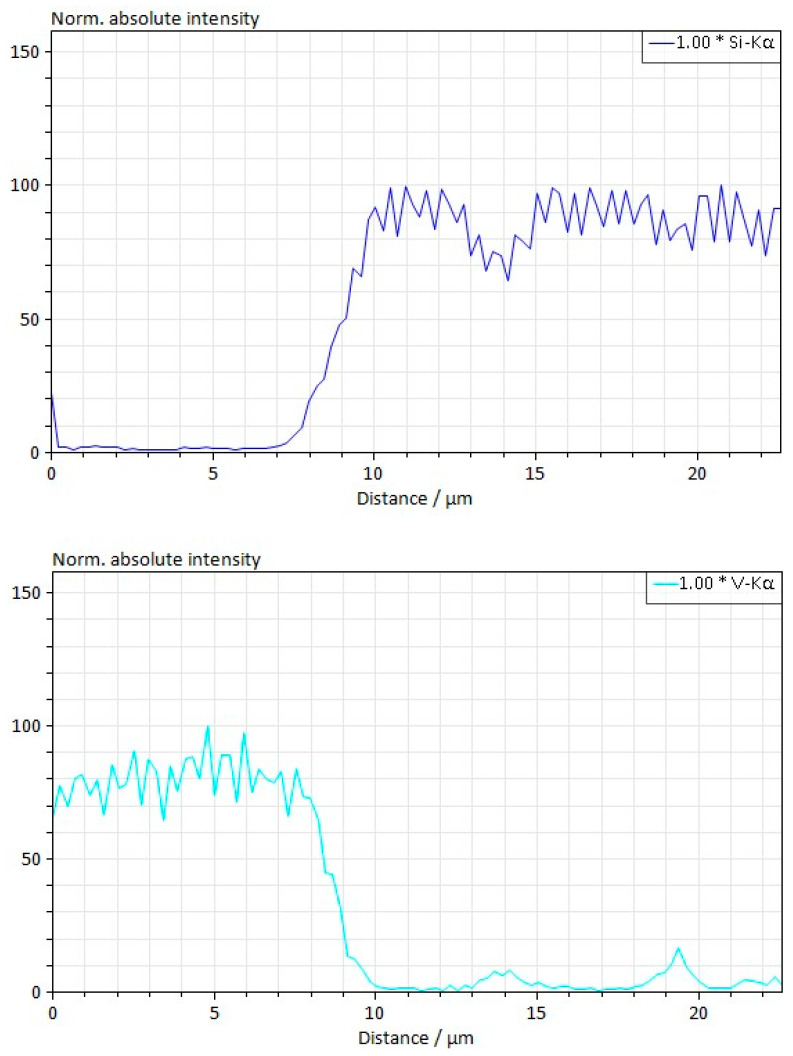
Zone of interaction between grains in the 60SiC–40VC composite obtained at a sintering temperature of 2000 °C, a pressure of 45 MPa, and a holding time of 30 min.

Studies have shown that during electrospark sintering at a pressure of 45 MPa, solid-state sintering in composites with an admixture of titanium carbide is better than in composites with an admixture of vanadium carbide due to increased interaction at the phase boundaries. The interaction zone increases from ~1.0 μm at the boundaries of silicon carbide grains and vanadium carbide grains (Figure 7). It increases to ~1.5 μm at the boundaries of silicon carbide grains and titanium carbide grains (Figure 8). It is suggested that this is due to a certain non-stoichiometric nature of TiC, which may enhance the interaction during sintering.

Table 2 presents the results of measuring the porosity *P*, crack resistance *K*_1*c*_, and hardness *H_v_* of the obtained composites.

As can be seen from Table 2, the introduction of TiC and VC additives decreases the porosity of composites. At the same time, the crack resistance of composites increases with increasing TiC and VC content. It can also be observed that the microhardness of SiC increases with the addition of TiC and VC. This can be explained by the high values of microhardness of the carbide additives used. As for the increase in the values of crack resistance with increasing TiC and WC content, it can be explained by the interaction at the phase boundaries and the strengthening of the composites as a whole.

## 5. Conclusions

The kinetics of electrospark sintering of SiC–TiC, SiC–VC composites at a pressure of 45 MPa at temperatures of 1900 and 2000 °C were investigated. The sintering kinetic constants *k* (characterizing the densification rate) and *n* (characterizing the densification process time) were determined.

At the first stage of the densification process, adding TiC and VC impurities at an amount of 20 vol.% to silicon carbide with a dispersion of 2 μm increases the densification rate by 1.3 and 1.1 times, respectively, and adding titanium carbide and vanadium carbide to silicon carbide in an amount of 40 vol.% increases the densification rate by 1.7 and 1.2 times, respectively.

It was revealed that at the second stage of the densification process, the adding of 40 vol.% Ti and V carbides to silicon carbide with a dispersion of 2 μm significantly increases densification: with optimal parameters of electrospark sintering, the maximum densification increases from ~70% in silicon carbide. In contrast, it increases to 99.9% in the 60SiC–40TiC composite and 91.2% in the 60SiC–40VC composite.

It was found that during electrospark sintering at a pressure of 45 MPa, solid-state sintering in composites with titanium carbide admixture is better than in composites with vanadium carbide admixture due to increased interaction at phase boundaries. The interaction zone increases from ~1.0 μm at the boundaries of silicon carbide grains and vanadium carbide grains to ~1.5 μm at the boundaries of silicon carbide grains and titanium carbide grains. The conducted studies allow us to conclude that to improve the process of compaction and the sintering of silicon carbide-based composites, it is necessary to use ultra-dispersive additives of titanium carbide and vanadium carbide as additives.

## Figures and Tables

**Table 1:** Kinetic constants *n* and *k* during electrospark sintering of SiC–TiC, SiC–VC composites.

No.	Composition, vol.%	Temperature, °C	Pressure, MPa	Stage 1		Stage 2		P, %
				*n*	*k*	*n*	*k*	
1	60% SiC, 40% TiC	1900	45	1.3	0.11	0.6	0.47	0.8
2	60% SiC, 40% TiC	2000	45	1.3	0.14	0.7	0.48	0.1
3	80% SiC, 20% TiC	2000	45	2.0	0.04	0.4	0.60	12.0
4	60% SiC, 40% VC	1900	45	1.8	0.01	0.5	0.38	14.3
5	60% SiC, 40% VC	2000	45	1.7	0.03	0.5	0.39	8.8
6	80% SiC, 20% VC	2000	45	–	–	–	–	25.0
7	100% SiC (2 mm)	2000	45	2.1	–	0.1	0.69	30.4

**Table 2:** Physical and mechanical properties of the obtained composites.

No.	Composition, vol.%	Sintering Parameters			*P*, %	*K*_1*c*_, MPa^1/2^	*H_v_*, GPa
		Temperature, °C	Pressure, MPa	Time, min.			
1	100% SiC (2 mm)	2000	45	30	30.3	2.9	2.9
2	80% SiC, 20% TiC	2000	45	30	12.0	4.3	6.6
3	60% SiC, 40% TiC	2000	45	30	0.1	5.7	21.5
4	80% SiC, 20% VC	2000	45	30	25.0	2.6	3.4
5	60% SiC, 40% VC	2000	45	30	8.8	5.2	13.7

## Data Availability

Data available on request due to privacy restrictions.
